# Nucleus accumbens shell small conductance potassium channels underlie adolescent ethanol exposure-induced anxiety

**DOI:** 10.1038/s41386-019-0415-7

**Published:** 2019-05-16

**Authors:** Lili Shan, Ewa Galaj, Yao-Ying Ma

**Affiliations:** 1 Behavioral Neuroscience Program, Department of Psychology, Binghamton University, State University of New York, Binghamton, NY 13902 USA; 2 Department of Pharmacology and Toxicology, Indiana University School of Medicine, 635 Barnhill Drive, Indianapolis, IN 46202 USA; 3 Stark Neurosciences Research Institute, Indiana University School of Medicine, Indianapolis, IN 46202 USA

**Keywords:** Anxiety, Striatum

## Abstract

Alcohol use typically begins in adolescence, increasing the likelihood of adult mental disorders such as anxiety. However, the cellular mechanisms underlying the consequences of adolescent alcohol exposure as well as the behavioral consequences remain poorly understood. We examined the effects of adolescent or adult chronic intermittent ethanol (CIE) exposure on intrinsic excitability of striatal medium-sized spiny neurons (MSNs) and anxiety levels. Rats underwent one of the following procedures: (1) light–dark transition (LDT) and open-field (OF) tests to evaluate anxiety levels and general locomotion; (2) whole-cell patch clamp recordings and biocytin labeling to assess excitability of striatal MSNs, as well as morphological properties; and (3) western blot immunostaining to determine small conductance (SK) calcium-activated potassium channel protein levels. Three weeks, but not 2 days, after CIE treatment, adolescent CIE-treated rats showed shorter crossover latency from the light to dark side in the LDT test and higher MSN excitability in the nucleus accumbens shell (NAcS). Furthermore, the amplitude of the medium afterhyperpolarization (mAHP), mediated by SK channels, and SK3 protein levels in the NAcS decreased concomitantly. Finally, increased anxiety levels, increased excitability, and decreased amplitude of mAHP of NAcS MSNs were reversed by SK channel activator 1-EBIO and mimicked by the SK channel blocker apamin. Thus, adolescent ethanol exposure increases adult anxiety-like behavior by downregulating SK channel function and protein expression, which leads to an increase of intrinsic excitability in NAcS MSNs. SK channels in the NAcS may serve as a target to treat adolescent alcohol binge exposure-induced mental disorders, such as anxiety in adulthood.

## INTRODUCTION

Alcohol is the most widely used substance of abuse and most people in the United States begin to use alcohol during adolescence [1, 2]. Adolescent alcohol drinking, which can be modeled in laboratory animals by chronic intermittent ethanol (CIE) exposure, is a serious public health concern, with 7.7 million individuals between the ages of 12–20 years reporting drinking alcohol within the past month [3]. Young people (5.1 million) reported binge drinking at least once in the past month and over 90% of alcohol consumed by underage drinkers is in the form of binge-drinking episodes [2]. This prevalence of binge alcohol drinking occurs at a critical period during development when the central nervous system is undergoing rapid adaptations in structure and function that could lead to vulnerability to mental disorders, such as anxiety. Epidemiological studies have shown that insidious alcohol use in adolescence predicts a vulnerability to mood disorders in adulthood [4].

The behavioral consequences, especially the prolonged effects in early adulthood, of adolescent alcohol exposure in the clinic cause substantial morbidity, but available treatments are limited. One reason is the lack of sufficient understanding about the neuroalterations induced by adolescent binge alcohol exposure and how these changes contribute to the associated mental disorders after a prolonged withdrawal period. Adolescence is a critical period with the concurrence of high vulnerability to the prolonged effects of CIE and a key stage of striatal development [5]. The striatum, including the nucleus accumbens (NAc) shell (NAcS), NAc Core (NAcC), and dorsolateral striatum (DLS), is a complex, multi-component structure possessing a ventromedial-to-dorsolateral gradient structurally and functionally [6]. Interestingly, in animal models, a striatum-anxiety link has emerged at both the circuit level (i.e., striatal innervation from the traditional anxiety-related brain regions) [7] and the behavioral level (e.g., open-field (OF) test, light–dark transition test) [8, 9].

The functional output of a brain region, by definition, is the action potential of the projecting neurons in that region [10], which is directly related to the intrinsic excitability of these neurons. Although increased excitability in the NAcC was reported after alcohol exposure starting during adolescence and continued until adult stage, it remains unclear how the intrinsic membrane excitability of the major striatal neuronal population, the projecting medium-sized spiny neurons (MSNs) [11], is affected by adolescent CIE exposure, especially after a prolonged withdrawal period. The excitability of MSNs can be affected by the amplitude of the action potential afterhyperpolarization (AHP), including both the medium AHP (mAHP), assumed to be mediated by small conductance (SK) calcium-activated potassium channels, and the fast AHP (fAHP), assumed to be mediated by large conductance (BK) calcium-activated potassium channels [12]. The present study was designed to explore the contribution of SK and BK potassium channels on MSN intrinsic excitability in three striatal subregions (NAcS, NAcC, and DLS) from rats 2 days or 3 weeks after CIE exposure compared with chronic intermittent water (CIW) in the adolescent vs. adult stage. Furthermore, we attempted to reverse the development-specific effects of CIE on the intrinsic excitability of MSNs, which may lead to a restoration of normal anxiety-like behavior levels.

## MATERIALS AND METHODS

More details are available in Supplementary Information.

### Experimental subjects

All procedures were performed in accordance with the United States Public Health Service Guide for Care and Use of Laboratory Animals, and were approved by the institutional Animal Care and Use committee at the State University of New York, Binghamton. Experiments were conducted on male Sprague–Dawley rats, bred in-house using breeders originally derived from Envigo, USA.

### CIE exposure

Our CIE procedure was adapted from a previous publication [13]. Briefly, adolescent (“Ado,” postnatal (P) 28–P47) or adult (“Adu,” P70–P89) rats received 4.0 g/kg intragastric administration of 25% (v/v) ethanol (CIE) or equivalent volume of water (CIW) once per day at ~9:00 a.m. in a 3-day on and 2-day off pattern as one cycle, repeated 4 times in total (20 days).

## RESULTS

### Increased anxiety-like behavior 3 weeks after adolescent but not adult CIE

**Fig. 1:**
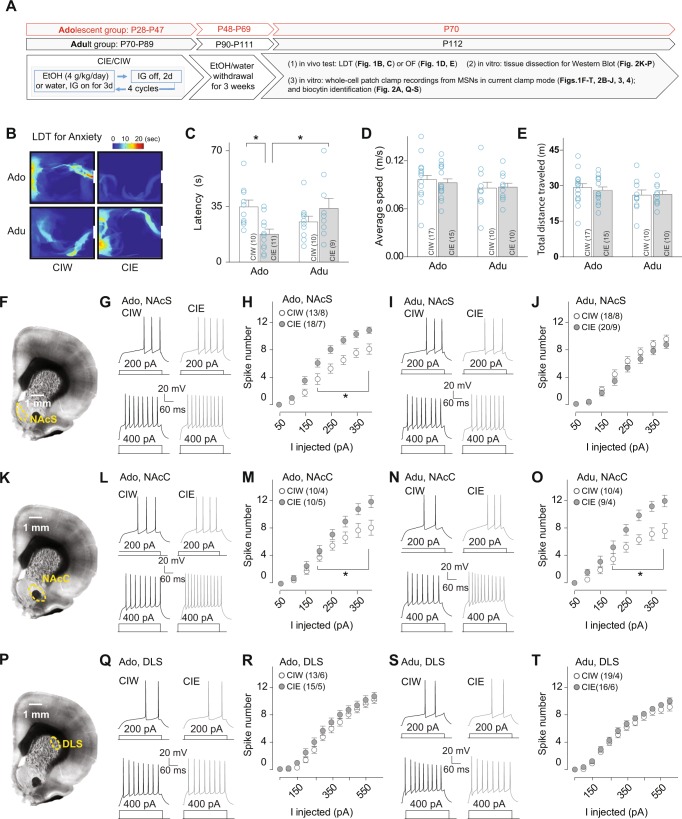
Evaluation of anxiety-like behavior and striatal MSN excitability 3 weeks after adolescent or adult treatment by CIW vs. CIE. **a** Experimental timeline for Fig. 1, Figs. 2–4. **b**–**e** Increased anxiety-like behavior 3 weeks after adolescent but not adult CIE. Representative maps in the light compartment of LDT test, surrounded by black lines (indicating the walls of light compartment) and white bars (indicating the tunnel to the dark compartment), on rats treated by CIW vs. CIE during adolescent (Ado) vs. adult (Adu) stage (**b**). Summarized results showing that relative to CIW group, CIE treatment in the adolescent stage specifically decreased the crossover latency from light to dark compartment, indicating a higher anxiety level in the early adult stage induced by adolescent CIE treatment (CIW/CIE × adolescent/adult interaction F_1,36_ = 9.9, *p* < 0.01) (**c**). Summarized results showing that average speed (**d**, CIW/CIE × adolescent/adult interaction F_1,48_ = 0.2, *p* = 0.66) and total distance traveled (**e**, CIW/CIE × adolescent/adult interaction F_1,48_ = 0.2, *p* = 0.65) were not affected by CIE treatment in either adolescent or adult stage. **f**–**j** Increased excitability of MSNs in the NAcS 3 weeks after adolescent, but not adult, CIE. Example DIC image of coronal section at a low magnification (through ×4 objective) showing the anatomical boundaries used to collect data from the NAcS (**f**). Example traces showing action potentials elicited by 200 and 400 pA current injections in NAcS MSNs from rats treated with CIW (left in each panel) and CIE (right in each panel) during the adolescent (**g**) or the adult (**i**) stage. Summarized data showing that excitability of NAcS MSNs is increased in rats with a CIE vs. CIW history during their adolescent stage (**h**, Ado::CIW/Ado::CIE × *I*_inj_ interaction, F_7203_ = 6.7, *p* < 0.01, cell based; F_7,91_ = 10.2, *p* < 0.01, animal based) but not adult stage (**j**, Adu::CIW/Adu::CIE × *I*_inj_ interaction, F_7252_ = 1.0, *p* = 0.44, cell based; F_7105_ = 1.1, *p* = 0.37, animal based). **k**–**o** Increased excitability of MSNs in the NAcC 3 weeks after both adolescent and adult CIE. Example DIC image of coronal section at a low magnification (through ×4 objective) showing the anatomical boundaries used to collect data from the NAcC (**k**). Example traces showing action potentials elicited by 200 and 400 pA current injections in NAcC MSNs from rats treated with CIW (left in each panel) and CIE (right in each panel) during the adolescent (**l**) or the adult (**n**) stage. Summarized data showing significant injected current-dependent differences in excitability of NAcC MSNs from rats treated with CIW vs. CIE during both their adolescent (**m**, Ado::CIW/Ado::CIE × *I*_inj_ interaction F_7126_ = 4.1, *p* < 0.01, cell based; F_7,49_ = 2.9, *p* = 0.01, animal based) and adult (**o**, Adu::CIW/Adu::CIE × *I*_inj_ interaction, F_7119_ = 3.7, *p* < 0.01, cell based; F_7,42_ = 2.4, *p* = 0.03, animal based) stage. **p**–**t** No changes in excitability of DLS MSNs 3 weeks after either adolescent or adult CIE. Example DIC image of coronal section at a low magnification (through ×4 objective) showing the anatomical boundaries used to collect data from the DLS (**p**). Example traces showing action potentials elicited by 200 and 400 pA current injections in DLS MSNs from rats treated with CIW (left in each panel) and CIE (right in each panel) during the adolescent (**q**) or the adult (**s**) stage. Summarized data showing no injected current-dependent difference in excitability of DLS MSNs from rats treated with CIW vs. CIE during their adolescent (**r**, Ado::CIW/Ado::CIE × *I*_inj_ interaction F_7182_ = 1.0, *p* = 0.41, cell based; F_7,63_ = 1.3, *p* = 0.27, animal based), or adult (**t**, Adu::CIW/Adu::CIE × *I*_inj_ interaction F_7231_ = 0.6, *p* = 0.79, cell based; F_7,56_ = 0.6, p = 0.75, animal based) stage. The animal number (i.e., *n* in **c**–**e**) or cell number/animal number (i.e., *m*/*n* in **h**, **i**, **m**, **o**, **r**, **t**) is shown in parentheses for each group. Data were analyzed by two-way ANOVA (**c**–**e**) or two-way ANOVA with repeated measures (**h**, **i**, **m**, **o**, **r**, **t**), followed by Bonferroni post test. *p < 0.05

Rats received CIW or CIE treatment during either adolescent or adult stages. To determine whether CIE affects the anxiety-like behavior level in a prolonged time fashion and whether this effect is developmentally regulated, 3 weeks after CIW or CIE, the light–dark transition (LDT) test was performed to evaluate the anxiety-like behavior level (Fig. 1a-c). We found that 3 weeks after oral gavage ethanol administration, the crossover latency in Ado::CIE group (16.4 ± 3.0 s) was significantly shorter than that in rats with a history of Ado::CIW (35.0 ± 4.0 s), Adu::CIW (25.3 ± 3.3 s), or Adu::CIE (31.6 ± 5.9 s). The specific effects of Ado::CIE on adult anxiety levels were also confirmed through OF test by comparing the percent time spent in the peripheral area of the OF (Ado::CIW, 79 ± 3%; Ado::CIE, 89 ± 2%; Adu::CIW, 80 ± 4%; Adu::CIE, 76 ± 4%; CIW/CIE × Ado/Adu interaction F_1,30_ = 4.9, *p* = 0.03). Furthermore, the potential impairment of general locomotion in rats treated by Ado::CIE was excluded, as no differences were found in average speed and total distance traveled in the OF test (Fig. 1d-e). Last, but not least, no difference of crossover latency between Ado::CIW (35.3 ± 5.0 s) vs. Ado::CIE (37.4 ± 4.9 s) groups, as well as their general locomotion, was detected 2 days after CIE treatment (Fig. S1B-D), demonstrating that the increased anxiety-like behavior occurs 3 weeks but not 2 days after Ado::CIE.

### Increased excitability of MSNs in the NAcS 3 weeks after adolescent CIE

To explore the potential neuronal substrates involved in the heightened anxiety-like behavior in rats with an adolescent but not adult CIE history, the intrinsic excitability of striatal MSNs was measured by whole-cell patch clamp in the NAcS, the NAcC, and the DLS 3 weeks after CIW or CIE exposure (timeline shown in Fig. 1a). A significant increase of injected current (denoted “*I*_inj_”)-dependent firing rate was detected in the NAcS from rats with an Ado::CIE history compared with those with an Ado::CIW history (Fig. 1g, h). No difference of firing rate was detected in the NAcS from rats with an adult history of CIW vs. CIE (Fig. 1i, j). In addition, the excitability of MSNs in the NAcS, increased 3 weeks after Ado:CIE, was not changed 2 days after Ado::CIE treatment (Fig. S1E, F). Interestingly, increases in excitability of MSNs in the NAcC were detected between CIW vs. CIE rats, treated either at the adolescent (Fig. 1l, m) or adulthood stage (Fig. 1n, o). No difference of injected current-dependent firing rate was detected in the DLS in rats treated with CIW vs. CIE in their adolescent (Fig. 1q, r) or adult stage (Fig. 1s, t). Considering that adolescent but not adult CIE increased anxiety-like behavior, the excitability changes in the NAcS, exclusively observed after Ado::CIE but not Adu::CIE, implicate the NAcS as the only region, among the three striatal subregions, which parallels the behavioral effects of CIE. Thus, the adolescent CIE procedure led to progressively increased anxiety-like behaviors and MSN excitability in the NAcS by the passage of the withdrawal period. The following experiments, to collect data 3 weeks after CIW/CIE treatment, were designed to specifically explore the potential mechanisms of the increased excitability of MSNs after CIE, which may provide a molecular substrate aimed at manipulating anxiety-like behaviors.

### Decreased electrophysiological function and expression of SK channels in NAcS MSNs from rats exposed to CIE during their adolescent stage

**Fig. 2:**
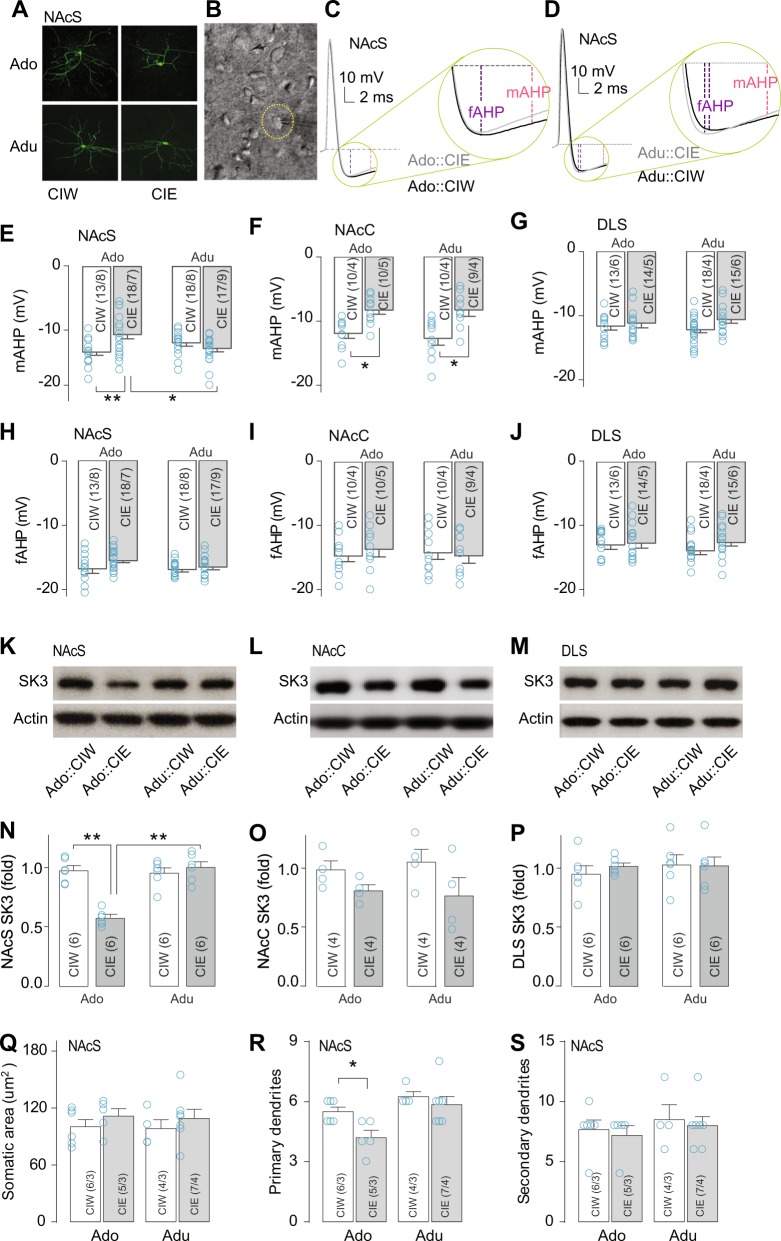
Electrophysiological, biochemical, and morphological assays associated with excitability changes in striatal MSNs. **a** Example confocal images of MSNs in the NAcS of rats 21 days after withdrawal from CIW or CIE treatment during adolescent or adult stage. **b** Example DIC image at a high magnification (through ×40 objective) showing an MSN in the NAcS patched with a micropipette. **c**–**j** Decreased mAHP in NAc MSNs from rats exposed to CIE during their adolescent stage. Example traces showing the fAHP and mAHP in NAcS MSNs from rats treated with CIW vs. CIE during their adolescent (**c**) and adult (**d**) stage. Summarized data showing that the mAHP (**e**, Ado/Adu × CIW/CIE interaction, F_1,62_ = 9.2, *p* < 0.01, cell based; F_1,28_ = 6.5, *p* = 0.02, animal based), but not fAHP (**h**, Ado/Adu × CIW/CIE interaction, F_1,62_ = 1.6, *p* = 0.21, cell based; F_1,28_ = 0.9, *p* = 0.35, animal based), was decreased in NAcS MSNs in rats pretreated with CIE during their adolescent stage relative to those treated with CIW during their adolescent stage or those treated with CIW or CIE during their adult stage. In NAcC MSNs in rats pretreated with CIE, either during their adolescent or adult stage, compared with those treated with CIW, mAHP was decreased (**f**, Ado/Adu × CIW/CIE interaction, F_1,35_ = 0.2, *p* = 0.65, cell based; F_1,13_ = 0.1, *p* = 0.73, animal based; Ado/Adu, F_1,35_ = 0.2, *p* = 0.67, cell based; F_1,13_ = 0.2, *p* = 0.67, animal based; CIW/CIE, F_1,35_ = 23.4, *p* < 0.01, cell based; F_1,13_ = 14.9, *p* < 0.01, animal based), but not fAHP (**i**, Ado/Adu × CIW/CIE interaction, F_1,35_ = 0.6, p = 0.44, cell based; F_1,13_ = 0.3, p = 0.62, animal based). Neither the amplitude of fAHP (**j**, Ado/Adu × CIW/CIE interaction, F_1,56_ = 0.6, *p* = 0.45, cell based; F_1,17_ = 0.8, *p* = 0.38, animal based) nor mAHP (**g**, Ado/Adu × CIW/CIE interaction, F_1,56_ = 0.9, *p* = 0.36, cell based; F_1,17_ = 1.0, *p* = 0.33, animal based) in DLS MSNs was affected in rats pretreated with CIE during their adolescent stage. **k**–**p** Decreased SK3 protein expression in the NAcS from rats exposed to CIE during their adolescent stage. Example western blotting bands showing SK3 protein levels in the NAcS (**k**), NAcC (**l**), and DLS (**m**) from rats treated by CIW vs. CIE during their adolescent and adult stage, respectively. Summarized results showing that decreased SK3 protein level in the NAcS from rats exposed to CIE during their adolescent but not adult stage (**n**, Ado/Adu × CIW/CIE interaction F_1,20_ = 30.1, *p* < 0.01) and the NAcC from rats exposed to CIE during both their adolescent and adult stage (**o**, Ado/Adu × CIW/CIE interaction F_1,12_ = 0.26, *p* = 0.61; CIW/CIE F_1,12_ = 5.0, *p* = 0.04; Ado/Adu F_1,12_ = 0.01, *p* = 0.92), but not in the DLS from rats with either adolescent or adult CIE history (**p**, Ado/Adu × CIW/CIE interaction F_1,20_ = 0.3, *p* = 0.61). **q**–**s** Morphological changes in NAcS MSNs. Summarized data showing the somatic area (**q**, Ado/Adu × CIW/CIE interaction, F_1,18_ = 0.1, *p* = 0.98, cell based) and the number of primary (**r**, Ado/Adu, F_1,18_ = 11.6, *p* < 0.01, cell based; CIW/CIE, F_1,18_ = 5.7, *p* = 0.03, cell based; interaction, F_1,18_ = 1.6, *p* = 0.22, cell based) and secondary dendrites (**s**, Ado/Adu × CIW/CIE interaction, F_1,16_ = 0.1, *p* = 0.99, cell based) per MSNs in the NAcS from rats with a history of CIW/CIE during adolescent or adult stage. The cell number/animal number (i.e., *m*/*n* in **c**–**j, q**–**s)** or the animal number (i.e., *n* in **n**–**p**) is shown in parentheses for each group. Data were analyzed by two-way ANOVA, followed by Bonferroni post test. *p < 0.05; **p < 0.01

Neuronal intrinsic excitability is tightly regulated by the amplitude of the AHP. It has been shown that decreased amplitude of AHPs leads to an increase in excitability [14]. Thus, we measured the amplitude of fAHP (i.e., the negative peak of the AHP, usually detected at ~10 ms after the onset of the action potential) and mAHP (i.e., the amplitude at 20 ms after the onset of the action potential), sampled after the first action potential elicited by +300 pA current injection (Fig. 2c, d, example traces). Compared with data from the CIW-treated group, decreased amplitude of mAHP was detected in NAcS MSNs from rats treated by Ado::CIE but not Adu::CIE (Fig. 2e). Consistent with the changes in excitability, decreased amplitude of mAHP was detected in NAcC MSNs from rats treated by both Ado::CIE and Adu::CIE (Fig. 2f). The amplitude of mAHP in DLS MSNs was not affected by either Ado::CIE or Adu::CIE treatment (Fig. 2g). No difference of fAHP between CIW vs. CIE was detected from rats treated during their adolescent or adult stage in MSNs from the NAcS (Fig. 2h), the NAcC (Fig. 2i), or the DLS (Fig. 2j). The fAHP and mAHP are mediated by BK and SK channels, respectively. Decreased amplitude of mAHP in the NAcS from Ado::CIE-treated rats suggested a downregulation of the channel protein levels in the NAcS from rats with a history of Ado::CIE. Immunoblotting results confirmed our assumption. SK3, as one of the major protein subtypes of striatal SK channels [15], was detected at a lower level in the NAcS from Ado::CIE-treated rats (Fig. 2k, n). Furthermore, decreased SK3 protein was detected in the NAcC from rats treated by both Ado::CIE and Adu::CIE (Fig. 2l, o). As expected, no difference of SK3 protein levels was detected in the DLS (Fig. 2m, p). Our results demonstrated the contribution of SK3 protein in regulating neuronal excitability; the increased excitability of striatal MSNs was always accompanied by decreased SK protein levels. Specifically, increased excitability and decreased SK3 protein levels were observed (1) in the NAcS 3 weeks after Ado::CIE but not Adu::CIE and (2) in the NAcC 3 weeks after both Ado::CIE and Adu::CIE.

### Morphological changes in MSNs of the NAcS

A subset of MSNs in the NAcS filled with biocytin (examples in Fig. 2a) was selected for detailed morphological analysis based on the quality of labeling and their somatic and dendritic field integrity. No significant somatic area change in these MSNs was detected (Fig. 2q in μm^2^: Ado::CIW, 100 ± 8; Ado::CIE, 111 ± 8; Adu::CIW, 98 ± 9; Adu::CIE, 109 ± 10). Interestingly, the number of primary, but not secondary, dendrites in the NAcS MSNs from rats with an adolescent CIE history was significantly decreased (Fig. 2r, primary dendrite # per MSN, 5.5 ± 0.2 in Ado::CIW, 4.2 ± 0.4* in Ado::CIE, 6.2 ± 0.3 in Adu::CIW, 5.6 ± 0.2 in Adu::CIE; Fig. 2s, secondary dendrite # per MSN, 7.7 ± 0.8 in Ado::CIW, 7.2 ± 0.8 in Ado::CIE, 8.5 ± 1.3 in Adu::CIW, 8.0 ± 0.8 in Adu::CIE). The branching rate of the primary dendrites, calculated by dividing the number of secondary dendrites by the number of primary dendrites, in the NAcS MSNs from rats with a history of adolescent CIE showed an increased trend (branches per primary dendrite, 1.4 ± 0.1 in Ado::CIW, 1.7 ± 0.1 in Ado::CIE, 1.3 ± 0.1 in Adu::CIW, 1.4 ± 0.1 in Adu::CIE), although the difference was not statistically significant. We assume that the decreased dendritic membrane size due to decreased primary dendrites in Ado::CIE rats was compensated by increased branching rate; therefore, the secondary dendrites are at similar levels among groups. This may explain why no significant differences in cell membrane capacitance (in pF, 116.0 ± 5.2 in Ado::CIW, 120.2 ± 5.0 in Ado::CIE, 114.9 ± 3.6 in Adu::CIW, 117.0 ± 4.7 in Adu::CIE) or input resistance (in MΩ, 237.3 ± 8.1 in Ado::CIW, 241.9 ± 6.7 in Ado::CIE, 234.4 ± 12.1 in Adu::CIW, 226.7 ± 9.8 in Adu::CIE) were detected among groups. Thus, the increased excitability of NAcS MSNs from Ado::CIE rats cannot be attributed to membrane-associated changes in input resistance.

### SK channel activator 1-EBIO-decreased MSN firing in the NAcS from rats exposed to CIE during their adolescent stage

**Fig. 3:**
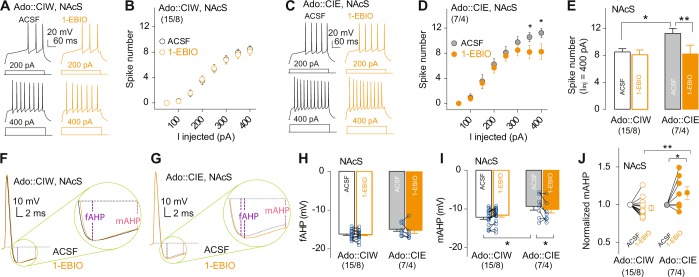
Activation of SK channels by 1-EBIO increased the mAHP amplitude in rats treated with CIE during adolescent stage, which reversed the effects of adolescent CIE on mAHP. **a**, **c** Example traces showing action potentials elicited by 200 and 400 pA current injections in NAcS MSNs in the ACSF vs. 1-EBIO (100 μM) from rats treated with CIW (**a**) or CIE (**c**) during the adolescent stage. **b**, **d** Summarized data showing an increased excitability of NAcS MSNs from rats with a history of CIE (**d**, ACSF/1-EBIO at 100 μM × *I*_inj_ interaction, F_7,98_ = 2.1, *p* = 0.04, cell based; F_7,21_ = 2.5, *p* = 0.04, animal based) but not CIW (**b**, ACSF/1-EBIO at 100 μM × *I*_inj_ interaction, F_7196_ = 0.21, *p* = 0.98, cell based; F_7,49_ = 0.15, *p* = 0.99, animal based) during their adolescent stage before vs. during application of 1-EBIO at 100 μM. **e** Summarized data of maximum spike numbers (*I*_inj_ = 400 pA) before and during bath application of 1-EBIO at 100 μM showing a specific increase of excitability in NAcS from rats treated with CIE during their adolescent stage (Ado::CIW/Ado::CIE × ACSF/1-EBIO at 100 μM interaction, F_1,21_ = 6.9, *p* = 0.02, cell based; F_1,10_ = 6.3, *p* = 0.03, animal based; Bonferroni post test, *p* < 0.05 between ACSF in Ado::CIE vs. any of the other three groups). **f**, **g** Example traces of the first action potential evoked by injecting current at +300 pA in NAcS MSNs from rats with a history of adolescent CIW (**f**) and CIE (**g**) before and during 1-EBIO at 100 μM. **h**, **i** Summarized results showing increased mAHP amplitude (**i**, Ado::CIW/Ado::CIE × ACSF/1-EBIO at 100 μM interaction, F_1,21_ = 4.5, *p* = 0.04, cell based; F_1,10_ = 5.0, *p* = 0.04, animal based; Bonferroni post test, *p* < 0.05 between ACSF in Ado::CIE vs. either ACSF in Ado::CIW or 1-EBIO at 100 μM in Ado::CIE) but no change in the fAHP (**h**, Ado::CIW/Ado::CIE × ACSF/1-EBIO at 100 μM interaction, F_1,21_ = 1.4, *p* = 0.25, cell based; F_1,10_ = 1.2, *p* = 0.30, animal based). **j** Summarized results showing 1-EBIO at 100 μM increased the normalized mAHP amplitude, by which the decreased mAHP amplitude in rats with an adolescent CIE history was reversed back to that in the CIW control group (Ado::CIW/Ado::CIE × ACSF/1-EBIO at 100 μM interaction, F_1,21_ = 4.8, *p* = 0.04, cell based; F_1,10_ = 5.2, *p* = 0.04, animal based; Bonferroni post test, *p* < 0.05 between 1-EBIO at 100 μM in Ado::CIE vs. either ACSF in Ado::CIE or 1-EBIO at 100 μM in Ado::CIW). The cell number/animal number (i.e., *m*/*n*) is shown in parentheses for each group. Data were analyzed by two-way ANOVA with repeated measures, followed by Bonferroni post test. *p < 0.05; **p < 0.01

In vitro pharmacological manipulations were done on brain slices dissected 3 weeks after adolescent CIE or CIW treatment (timeline shown in Fig. 1a). 1-EBIO at 100 and 300 μM doses, selected according to previous publications [16–18], was bath applied to the striatum-containing slices during patch clamp recordings. Relative to the higher dose (data not shown), 1-EBIO at 100 μM selectively decreased intrinsic excitability of MSNs in the NAcS from rats with a history of CIE during their adolescent stage, although 1-EBIO at both doses produced significant effects on MSNs. Decreased firing rate by 1-EBIO at 100 μM was detected in an injected current-dependent manner in rats treated by CIE during their adolescent stage (Fig. 3c, d). No differences in firing rates between ACSF vs. 1-EBIO at 100 μM were observed in the rats treated by CIW during their adolescent stage (Fig. 3a, b). Statistical analysis by comparing the maximal firing rate (i.e., evoked by 400 pA injection current) confirmed that 1-EBIO at 100 μM selectively downregulates the firing rate in rats with a CIE history during their adolescent stage to a similar level as in the CIW group (Fig. 3e). The downregulation of 1-EBIO on MSN excitability was accompanied by increased amplitudes of mAHPs (Fig. 3g, i), but no significant changes of fAHP were observed (Fig. 3f–h), suggesting specific effects of 1-EBIO on the SK channel-mediated mAHP. Further statistical analysis of the normalized mAHP amplitude shows that 1-EBIO at 100 μM selectively decreased the mAHP in rats with a CIE history (Fig. 3j). Altogether, our data demonstrated that the adolescent CIE treatment-increased intrinsic excitability can be reversed to control levels by selectively activating SK channels in the NAcS, through which mAHP amplitude was increased.

### SK channel blocker apamin-induced MSN firing in the NAcS from rats exposed to CIW during their adolescent stage

**Fig. 4:**
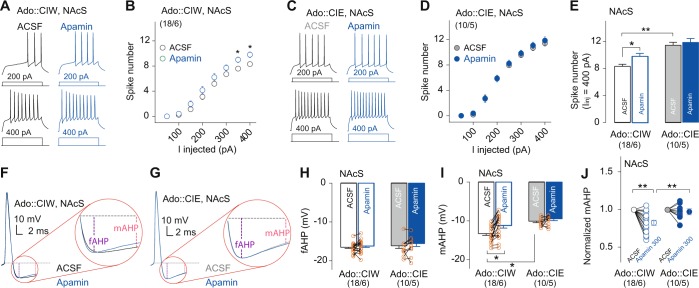
Blockade of SK channels by apamin decreased the mAHP amplitude in rats treated with CIW during the adolescent stage, which mimicked the effects of adolescent CIE on mAHP. **a**, **c** Example traces showing action potentials elicited by 200 and 400 pA current injections in NAcS MSNs in the ACSF vs. apamin (300 nM) from rats treated with CIW (**a**) and CIE (**c**) during the adolescent stage. **b**, **d** Summarized data showing an increased excitability of NAcS MSNs from rats with a history of CIW (**b**, ACSF/apamin at 300 nM × *I*_inj_ interaction, F_7238_ = 2.6, *p* = 0.01, cell based; F_7,35_ = 2.3, *p* = 0.04, animal based) but not CIE (**d**, ACSF/apamin at 300 nM × *I*_in_ interaction F_7126_ = 0.3, *p* = 0.97, cell based; F_7,28_ = 0.4, *p* = 0.89, animal based) during their adolescent stage. **e** Summarized data of spike number (*I*_inj_ = 400 pA) before and during bath application of apamin (300 nM) showing a specific increase of excitability in NAcS from rats treated with CIW during their adolescent stage (Ado::CIW/Ado::CIE × ACSF/apamin at 300 nM interaction, F_1,26_ = 6.9, *p* = 0.01, cell based; F_1,9_ = 6.2, *p* = 0.03, animal based; Bonferroni post test, *p* < 0.05 between ACSF in Ado::CIW vs. either apamin at 300 nM in Ado::CIW or ACSF in Ado::CIE). **f**, **g** Example traces of the first action potential evoked at *I*_inj_ = 300 pA in NAcS MSNs from rats with a history of adolescent CIW (**f**) and CIE (**g**) before and during apamin at 300 nM. **h**, **i** Summarized results showing apamin at 300 nM increased mAHP amplitude (**i**, Ado::CIW/Ado::CIE × ACSF/apamin at 300 nM interaction, F_1,26_ = 6.3, *p* = 0.02, cell based; F_1,9_ = 5.8, *p* = 0.04, animal based; Bonferroni post test, *p* < 0.05 between ACSF in Ado::CIW vs. either ACSF in Ado::CIE or apamin at 300 nM in Ado::CIW) but no significant effects were detected on the fAHP amplitude (**h**, Ado::CIW/Ado::CIE × ACSF/apamin at 300 nM interaction, F_1,26_ = 0.1, *p* = 0.83, cell based; F_1,9_ = 0.2, *p* = 0.67, animal based) in adolescent CIE- vs. CIW rats. **j** Summarized results showing apamin at 300 nM decreased the mAHP amplitude in adolescent CIW rats (Ado::CIW/Ado::CIE × ACSF/apamin at 300 nM interaction, F_1,26_ = 5.7, *p* = 0.02, cell based; F_1,9_ = 5.6, *p* = 0.04, animal based; Bonferroni post test, *p* < 0.05 between apamin at 300 nM in Ado::CIW *vs*. either ACSF in Ado::CIW or apamin at 300 nM in Ado::CIE). It is observed that the adolescent CIE effects on the amplitude of mAHP were mimicked in the CIW control group. The cell number/animal number (i.e., *m*/*n*) is shown in parentheses for each group. Data were analyzed by two-way ANOVA with repeated measures, followed by Bonferroni post test. *p < 0.05; **p < 0.01

We performed additional in vitro pharmacological manipulations on brain slices dissected 3 weeks after adolescent CIE or CIW treatment using the SK channel blocker apamin at 100 and 300 nM, respectively. Apamin doses were selected according to previous publications [16, 19]. Relative to the lower dose (data not shown), apamin at 300 nM significantly increased intrinsic excitability of MSNs and selectively targeted the mAHP but had no effects on the fAHP in NAcS from rats with a history of CIW treatment during their adolescent stage. Increased firing rate by apamin at 300 nM was detected in an injected current-dependent manner in rats treated by CIW (Fig. 4a, b) but not CIE (Fig. 4c, d) procedure during their adolescent stage. Statistical analysis by comparing the firing rate at 400 pA injection current confirmed that apamin at 300 nM selectively upregulated the firing rate in rats with a CIW history during their adolescent stage comparable to the level observed in the CIE group (Fig. 4e). The upregulation by apamin of MSN excitability was accompanied by decreased amplitude of mAHPs (Fig. 4f, i), but no significant changes of fAHP were observed (Fig. 4f–h), suggesting specific effects of apamin at 300 nM on the SK channel-mediated mAHP. Further statistical analysis of the normalized mAHP amplitude shows that apamin at 300 nM selectively decreased the mAHP in rats with a CIW history (Fig. 4j). Thus, our data demonstrate that the adolescent CIE treatment-increased intrinsic excitability can be mimicked in the control group by selectively blocking the SK channel in the NAcS, through which mAHP amplitude was decreased.

### Attenuated and enhanced anxiety-like behavior by activation and blockade of SK channel through microinjection of 1-EBIO and apamin, respectively

**Fig. 5:**
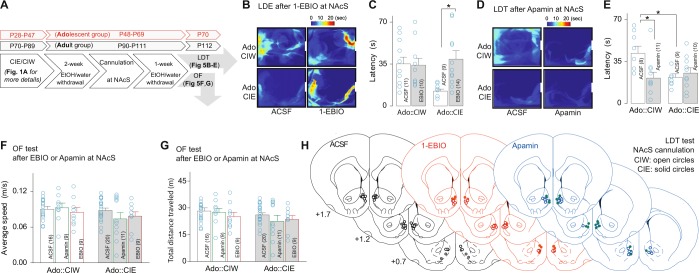
Adolescent CIE-induced anxiety-like behavior can be blocked by SK channel agonist 1-EBIO and mimicked by SK channel antagonist apamin microinjected into the NAcS. **a** Experimental timeline. **b** Example LDT maps in the light compartment, surrounded by black lines (indicating the walls of light compartment) and white bars (indicating the tunnel to the dark compartment) from rats with a history of CIW vs. CIE during their adolescent stage, treated with microinjections of ACSF vs. 1-EBIO (324 ng in 0.5 μL per injection site at a concentration of 4 mM) in the NAcS 10 min before test. **c** Summarized results showing 1-EBIO specifically increased the crossover latency in rats with a history of adolescent CIE treatment, by which their latency was reversed back to a similar level as in the CIW control group (Ado::CIW/Ado::CIE × ACSF/1-EBIO interaction F_1,40_ = 6.1, *p* = 0.02). **d** Example LDT maps from rats with a history of CIW vs. CIE during their adolescent stage, treated with microinjections of ACSF vs. apamin (2 ng in 0.5 μL, per injection site at a concentration of 2 μM) in the NAcS 10 min before the test. **e** Summarized results showing apamin specifically decreased the crossover latency in rats with a history of adolescent CIW treatment, by which their latency reaches a level similar to the adolescent CIE group Ado::CIW/Ado::CIE × ACSF/apamin interaction F_1,34_ = 12.6, *p* < 0.01. **f**, **g** Summarized results showing that, compared with ACSF, neither 1-EBIO nor apamin affected the speed (**f**, Ado::CIW/Ado::CIE × ACSF/1-EBIO interaction F_2,68_ = 0.62, *p* = 0.55) or the total distance traveled (**g**, Ado::CIW/Ado::CIE × ACSF/1-EBIO/apamin interaction F_2,68_ = 0.61, *p* = 0.54) during the OF test. **h** Diagrams of coronal slices showing the cannulation sites in the NAcS from rats used for LDT test. The animal number (i.e., *n*) is shown in parentheses for each group. Data were analyzed by two-way ANOVA, followed by Bonferroni post test. *p < 0.05

To examine the in vivo effects of pharmacological manipulations of the NAcS SK channels on the adolescent CIE-induced anxiety-like behavior 3 weeks after adolescent CIW or CIE treatment, the crossover latency from the light to the dark chamber in the LDT test was measured 10 min after microinjections of 1-EBIO (324 ng per injection site at a concentration of 4 mM) and apamin (2 ng per injection site at a concentration of 2 µM), respectively (timeline shown in Fig. 5a). 1-EBIO selectively increased the crossover latency in rats with an adolescent CIE history but had no effect on those with an adolescent CIW history (Fig. 5b, c). Moreover, apamin selectively decreased the crossover latency in rats with an adolescent CIW history but had no effect on those with an adolescent CIE history (Fig. 5d, e). No effects on the average speed (Fig. 5f) or total distance traveled (Fig. 5g) were detected after microinjection of 1-EBIO or apamin into the NAcS, suggesting neither in vivo pharmacological manipulation affected general locomotion in rats. Finally, bilateral microinjections of 1-EBIO or apamin at the same doses into the DLS did not affect the crossover latency in rats treated by CIW or CIW at their adolescent stage (Fig. S2), indicating the pharmacological effects of SK channel agonists or antagonists are brain-region specific.

## DISCUSSION

Two aspects of the affective effects of binge drinking are worth emphasizing in rodent models. First, we found that binge ethanol exposure increases anxiety-like behavior levels in a developmentally regulated manner, i.e., adolescent, but not adult binge exposure-heightened anxiety-like behaviors, which is consistent with recent data from a mouse CIE model [20]. Despite the age-related differences, adult rodents have also been reported as susceptible as adolescents to the consequences of CIE [21, 22]. However, potential caveats should be noted, such as delayed starting point (P120) of adult CIE treatment and short withdrawal period (i.e., 12 days) [22]. Second, we detected the increased anxiety-like behavior level after a prolonged withdrawal period from binge ethanol exposure, which is consistent with previous reports [23–25]. Interestingly, both decreased [26] and increased [27] anxiety-like behavior levels have been observed 1–2 days after adolescent CIE procedure. Moreover, acute ethanol exposure during adolescence produced an acute decrease of social anxiety-like behavior levels [28]. Thus, affective consequences of ethanol exposure depend, at least partially if not fully, on subjects’ age (e.g., adolescent vs. adult), exposure pattern (e.g., chronic vs. acute), and withdrawal period (e.g., acute vs. prolonged). The increased anxiety-like behavior after adolescent CIE could be either persistently or progressively increased with the passage to the withdrawal period. A very recent report from a mouse CIE model supports the latter (i.e., an incubation of anxiety-like behavior after adolescent CIE) [20], which is consistent with our current results.

Studies on anxiety, one of the typical affective consequences observed after adolescent CIE exposure, has primarily focused on the amygdala [29], bed nucleus of the stria terminalis (BNST) [30], hippocampus [31], and prefrontal cortex (PFC) [32]. However, the potential link between adolescent CIE-induced anxiety-like behaviors and the striatum has been substantially supported by data from our lab and others as follows. First, the striatum communicates with diverse components of the classical neural network of anxiety. The amygdala and the PFC project to the striatum directly and the striatum communicates with the hippocampus and BNST bi-directionally, positioning the striatum as an integral and central part of the anxiety circuit [7]. Second, adolescence is a key stage of striatal development. Striatal neurons and circuits display maturational changes during adolescence, and not childhood, unlike most of the other brain regions [33]. Third, high sensitivity of striatal MSNs to adolescent CIE was detected. The intrinsic excitability of MSNs has been considered as one of the two factors that determine the striatal output besides synaptic transmission. Significant increase in the intrinsic excitability of MSNs was detected in the NAcS (our current data) and the NAcC [15]. Fourth, striatal alterations have been detected in subjects showing high vulnerability to anxiety. Besides functional alterations detected in the current study, morphological changes have been also detected in the striatum [34]. In addition to the above arguments, the NAcS, as the major component in the ventromedial striatum, is expected to be more involved in anxiety, given its importance in emotional processes. It is worth noting that when looking at responses of MSNs we found no evidence that we were dealing with two different cell populations. Responses were pretty homogeneous in all MSNs. Although one could have expected divergent responses based on previous literature, in the present study this was not the case. However, we acknowledge that the use of animals expressing green fluorescent protein in D1 and D2 neurons would be required to confirm our findings.

A reduced SK channel function has been identified in several brain regions from ethanol-treated animals. Ethanol exposure reduced apamin-sensitive SK currents in CA1 pyramidal neurons in the hippocampus [35] and Ventral Tegmental Area dopamine neurons in the midbrain [36]. More related to the current findings, reduced SK currents were detected in the NAcC from rats withdrawn from ethanol self-administration starting during adolescence and continued until adulthood [15]. Although the manner of ethanol administration is one of the potential factors leading to unique neuronal adaptations, the developmental stage is hypothesized as the primary factor leading to specific neuroplasticity. Relative to the parallel control group with a CIW history, rats with a CIE history during their adolescent stage showed higher vulnerability to anxiety, but the rats treated by CIE procedure during their adult stage demonstrated similar anxiety-like behavior levels as the control group. Thus, the neuronal substrate we were looking for to rescue the adolescent CIE-induced anxiety-like behavior should be the adaptations occurring in rats with an adolescent CIE history only but will be lacking in rats with an adult CIE history. The adaptations of SK channels observed in the NAcS in the current studies perfectly match these criteria. Furthermore, lack of changes in SK channels in the DLS excluded the alternative explanation that the reduction of SK current is a nonspecific effect in the brain from rats withdrawn from an adolescent CIE treatment.

We found that low expression level of SK channel protein was associated with low sensitivity to the SK channel blocker but high sensitivity to SK channel activator, as observed in NAc MSNs from the Ado::CIE group. However, high levels of SK channel protein were associated with high sensitivity to a SK channel blocker but low sensitivity to a SK channel activator, as observed in NAc MSNs from the Ado::CIW group. Thus, we hypothesize a homeostatic interplay between SK protein levels and sensitivity to channel modulators.

The downregulation of SK channels after prolonged withdrawal from adolescent ethanol exposure could be an acute effect of adolescent CIE treatment, which is persistent during the whole withdrawal period, or accumulated chronic effects, which are incubated during the withdrawal period. Our data showed no changes of MSN excitability from rats 2 days after CIE::Ado, indicating an incubated SK protein level by the passage of the withdrawal period. Another alternative explanation for the low SK channel function in adult rats with an adolescent CIE history is that there is a development-dependent increase of SK channel function in the NAcS and the adolescent ethanol exposure could maintain the SK channel function at a low level, in a similar temporal course as the behavioral observations of persistence of typical adolescent characteristics into adulthood. That is, after adolescent exposure to ethanol, SK channel functional level during adolescence continues to be expressed at a low level until adulthood, weeks after termination of the adolescent exposure period. The concept that adolescent CIE history results in the retention of certain phenotypes from adolescence into adulthood apparently suggests that similar findings would not emerge from ethanol exposure at a time when the adolescent phenotype is no longer evident (i.e., adulthood), which matches our data showing no difference between adult CIE vs. adult CIW groups at both behavioral level, as measured by LDT test, and molecular level, measured by the function and protein expression of SK channels in the NAcS. A variety of studies showed retention of adolescent characteristics, including attenuated ethanol conditioned taste aversion, increased motivation for ethanol rewarding effects, elevated ethanol consumption, into adulthood after Ado::CIE [1]. Further exploration of SK channel functional contribution to the mAHP as well as its protein expression in the NAcS at different developmental stages spanning adolescence into adulthood will address this hypothesis of adolescent retention more directly.

The current study first demonstrated that, consistent with clinical observations, laboratory rodents also display a late-onset, negative effect, i.e., increased anxiety-like behavior, after adolescent, but not adult, binge alcohol exposure. Further exploration of the underlying neuronal and molecular substrates in brain slices showed that in rats with an adolescent CIE history, the intrinsic excitability of MSNs in the NAcS was selectively increased, at least partially by downregulated function and protein levels of SK channels in the NAcS. Corresponding to our in vitro pharmacological manipulations showing the prolonged effects of adolescent CIE can be reversed by activation of SK channels and mimicked by blockade of SK channels, our in vivo pharmacological manipulations showed that the anxiety-like behavior induced by adolescent CIE treatment can be reversed by 1-EBIO in the adolescent CIE group or mimicked in the adolescent CIW group by apamin. Bidirectional modification of intrinsic excitability suggested a causal relationship between SK channel functional levels to the prolonged mental effects of adolescent CIE treatment. Selective effects of activating or blocking SK channels on in vivo and in vitro measurements indicate that clinical treatment, i.e., significant anxiolytic effects with minimal side effects, can be reached by optimizing the dose of SK channel activator. Food and Drug Administration-approved drugs, such as chlorzoxazone, can activate SK channels in a similar manner as 1-EBIO. Our study provides insights into a way to prevent and treat adolescent alcohol exposure-induced mental disorders by improving SK channel function.

## Funding and disclosure

This work was supported by NIH grants (P50AA017823, R01AA025784, and T32AA025606) and Brain & Behavior Research Foundation grant #24989. The authors declare no competing interests.

## Supplementary information

Supplemental Figure 1

Supplemental Figure 2

Nucleus Accumbens Shell Small Conductance Potassium Channels Underlie Adolescent Ethanol Exposure-Induced Anxiety
