# Supplementary figures and images for: Nucleus accumbens shell small conductance potassium channels underlie adolescent ethanol exposure-induced anxiety
Source: Neuropsychopharmacology. 2019 May 16;44(11):1886–95. doi: 10.1038/s41386-019-0415-7 (PMC6784903; doi:10.1038/s41386-019-0415-7)

Fig. S1

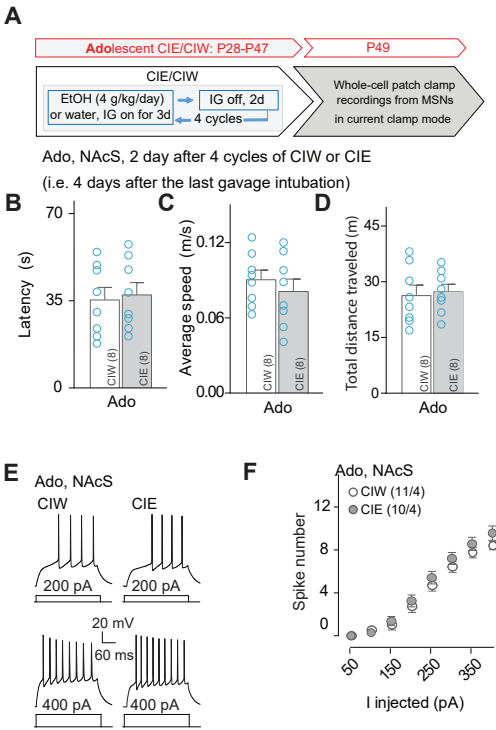

Supplement: Supplementary file 1 — Supplemental Figure 1 [file 41386_2019_415_MOESM1_ESM.pdf]

Fig. S2

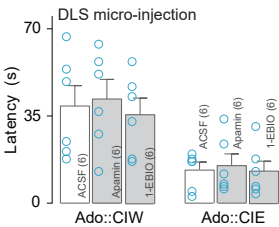

Supplement: Supplementary file 2 — Supplemental Figure 2 [file 41386_2019_415_MOESM2_ESM.pdf]
