# Supplementary material for: Nucleus accumbens shell small conductance potassium channels underlie adolescent ethanol exposure-induced anxiety
Source: Neuropsychopharmacology. 2019 May 16;44(11):1886–95. doi: 10.1038/s41386-019-0415-7 (PMC6784903; doi:10.1038/s41386-019-0415-7)
Supplement: Supplementary file 3 — Nucleus Accumbens Shell Small Conductance Potassium Channels Underlie Adolescent Ethanol Exposure-Induced Anxiety [file 41386_2019_415_MOESM3_ESM.docx]

**Nucleus Accumbens Shell Small Conductance Potassium Channels**

**Underlie Adolescent Ethanol Exposure-Induced Anxiety**

**SUPPLEMENTARY MATERIALS AND METHODS**

**Experimental Subjects:** With the day of birth being deemed as postnatal day (P) 0, rats were allowed to develop normally until P21-23 when animals were weaned and pair-housed in standard Plexiglas bins unless receiving cannula implantation procedure. Rats were maintained on a 7 AM / 7 PM light / dark schedule with *ad libitum* access to food and water. Rats were allowed 5-7 days to acclimate to colony conditions and handled to habituate them to human contact prior to experimentation. A total of 290 rats were used but 18 rats were excluded. Specifically, 2 rats were excluded before data collection because of complications after gavage intubation, 3 rats were excluded before data collection because of surgery-associated low body temperature, 2 rats were excluded during data collection because of clogged cannula or inaccurate injection volume into the NAcS,  5 rats were excluded after data collection because misplacement of cannulas (found during preparation of brain slices), 3 rats were excluded after data collection as outliers during LDT test and 3 more rats as outliers during OF test by eliminating any points not within two standard deviations of the group mean value.

**Evaluation of blood ethanol concentration:** Blood samples were taken from the tail vein in a separate cohort for the analysis of blood ethanol concentrations (BECs) 30 min after the last binge administration in Cycles 2 and 4 of the CIW or CIE. Blood samples were maintained at −80 °C until analysis. BECs were assessed via headspace gas chromatography using a Hewlett Packard (HP) 5890 series II Gas Chromatograph (GC) and procedures in standard use in our alcohol research center, the Developmental Exposure Alcohol Research Center [1,2]. Our data show that, in both Ado and Adu groups, CIE treatment significantly increased BECs by the end of cycle 2 (Ado::CIW, 11.5±0.5; Adu::CIW, 11.0±0.4; Ado::CIE, 164.7±9.5; Adu::CIE, 150.1±16.5) and cycle 4 (Ado::CIW, 13.7±0.8; Adu::CIW, 12.3±0.9; Ado::CIE, 203.4±10.1; Adu::CIE, 178.9±12.0). These concentrations are in the binge range (defined as >80 mg/dL by the NIAAA).

**Open field (OF) test:** Custom-crafted, black Plexiglas OF testing chamber was 90 cm for both side-widths x 50cm in height [3] with a floor illumination at ~60-80 lux. Center area was defined as 45 cm x 45 cm in the center. Rats were placed in the peripheral area facing one of the chamber’s sidewalls and then allowed to freely explore the chamber. Total testing time was 5 min.

**Light dark transition (LDT) test:** Custom-crafted, Plexiglas LDT testing chamber was composed of two different compartments, light side (50 cm in width x 40cm in depth x 30cm in height, 400 lux on floor surface) and dark side (40 cm in width x 40cm in depth x 30cm in height), which shared a sidewall with an entrance (7.5 cm x 7.5 cm). Rats were placed in the light side facing the sidewall of the light compartment opposite to the entrance. Then rats were allowed to freely explore the testing chamber. Testing sessions ended once the rats crossed over the entrance toward the dark compartment. Cross-over latency was used as an index inversely correlated to the anxiety level.

**Cannulation**: 2 weeks after CIE or CIW treatment, rats (n=74) were anesthetized with a ketamine/xylazine mixture (50/5 mg/kg, i.p.) and placed in a stereotaxic apparatus (KOPF, CA, USA). The coordinates used to target the NAcS were (in mm): +1.55 (AP), ±0.6 (ML) and -6.0 (DV, from the skull surface). Guide cannulae (26 G) were anchored to the skull with sterile stainless steel screws and dental cement. After surgery, a stainless steel obturator (0.4 mm in outer diameter) was inserted into the guide in order to prevent cannula occlusion. The obturator was removed and replaced every day during the 7-day recovery period.

**Microinjection into the NAcS** The obturator was removed and an infusion cannula (33 G) was inserted, extending 1.5 mm beyond the tip of the guide cannula into the NAcS. Bilateral microinjections of ACSF, 1-ethyl-2-benzimidazolinone (EBIO) (Alomone Labs, Jerusalem, Israel; 324 ng/0.5 µl per site at a concentration of 4 mM) or apamin (Alomone Labs, Jerusalem, Israel; 2 ng/0.5 µl per site at a concentration of 2 µM) were administered into the NAcS. These *in vivo* doses were chosen based on previous publications [4,5]. The injection was performed using an infusion pump (0.1 µl/min) while the rat was freely moving in the home cage. The infusion cannula was left in place for an additional 5 minutes to allow for drug diffusion. The obturator was then replaced, and rats were tested 10 min later.

**Brain slice whole-cell patch clamp recordings:** Standard procedures were used for preparing slices and whole-cell patch clamp recordings as detailed in our previous publications [6-8]. Before sacrifice, the rats were anesthetized with isoflurane and subsequently transcardially perfused with 4°C cutting solution (in mM: 135 *N*-methyl-D-glucamine, 1 KCl, 1.2 KH_2_PO_4_, 0.5 CaCl_2_, 1.5 MgCl_2_, 20 choline-HCO_3_, 11 glucose, pH adjusted to 7.4 with HCl, and saturated with 95% O_2_ /5% CO_2_). The rat was decapitated, and then the brain was removed and glued to a block before slicing using a Leica VT1200s vibratome in 4°C cutting solution. Coronal slices of 250 µm thickness were cut such that the preparation contained the signature anatomical landmarks (e.g., the anterior commissure and the corpus callosum) that clearly delineate the striatal subregions. After allowing at least 1 hr for recovery, slices were transferred from a holding chamber to a submerged recording chamber where it was continuously perfused with oxygenated ACSF maintained at 30 ± 1°C.

Standard whole-cell current- or voltage-clamp recordings were obtained with a MultiClamp 700B amplifier (Molecular Devices), filtered at 3 kHz, amplified 5 times, and then digitized at 20 kHz with a Digidata 1550A analog-to-digital converter (Molecular Devices). The recording electrodes (3-5 MΩ) were filled with (in mM): 108 KMeSO_3_, 20 KCl, 0.4 K-EGTA, 10 Hepes, 2.5 Mg-ATP, 0.25 Na-GTP, 7.5 phosphocreatine (Na2), 1 L-glutathione, 2 MgCl_2_, pH 7.3. The recording bath solution contained (in mM): 119 NaCl, 2.5 KCl, 2.5 CaCl_2_, 1.3 MgCl_2_, 1 NaH_2_PO_4_, 26.2 NaHCO_3_, and 11 glucose, saturated with 95% O_2_ / 5% CO_2_ at 30 ± 1°C. Details for whole-cell patch clamp recordings can be found in one of our previous publications [8]. In brief, MSNs in the NAcS, NAcC and DLS were located using the corpus callosum, anterior commissure and the Islands of Calleja as landmarks and recorded in coronal slices. Cells were patched in voltage clamp mode and held at −70 mV. Cell membrane capacitance (Cm), input resistance (Rm) and time constant (τ) were calculated by applying a depolarizing step voltage command (5 mV) and using the membrane test function integrated in the pClamp10 software. Then recordings were switched to current clamp mode. Resting membrane potential was adjusted to -80 mV through injecting a positive current (50 - 100 pA) and then intrinsic excitability was examined using a series of depolarizing current pulses and by constructing input-output (I-O) functions.

**Western blotting**

Procedures were adapted from our previous publications [9,10]. Animals were sacrificed to extract the whole brain, flash frozen and stored in -80 °C until processing. Tissue punches were performed from DLS and NAcS under -25 °C. Samples were homogenized in RIPA buffer (EMD Millipore) and immediately centrifuged at 12,000 g for 20 min at 4 °C. Supernatants were collected and diluted with 2x Tris-glycine SDS loading buffer (Thermo Fisher) containing 50 mM Dithiothreitol and samples were denatured for 10 min at 85 °C. Twenty micrograms of each sample were loaded respectively using 8%~16% Tris-glycine gel (Thermo Fisher) with Tris-glycine running buffer (Thermo Fisher). IBlot dry blotting system and PVDF membrane were used for protein transfer and then incubated in 0.05% Tween tris-buffered saline (TBST) containing 5% non-fat dried milk for 1 h at room temperature. The membrane was then incubated 2 h at room temperature with primary antibodies (SK3, 1:500, Alomone labs; Actin, 1:10,000, EMD Millipore) in TBST and then washed three times for 15 min in TBST before 1 h secondary antibody incubation (Goat anti-rabbit IgG, 1:10,000, Thermo Fisher; Goat anti-mouse IgG, 1:20,000, Thermo Fisher). The antigen-antibody peroxidase complex was then finally detected by SuperSignal™ West Femto Chemiluminescent Substrate (Thermo Scientific™, Cat#: PI34096) according to the manufacturer's instructions and acquired the band images using Fluor Chem E System of Protein Simple and then analyzed the intensity of bands through NIH Image J System. The signal intensity of the corresponding Actin band corrected the signal intensity of each band of the SK3 channel protein. The values are presented as a percentage of the control.

**Single-Cell Staining, Confocal Imaging and Morphological Measurements:** As described in one of our publications [8], striatal slices containing biocytin-filled cells were fixed with 4% paraformaldehyde in 0.1 M phosphate buffer, pH 7.4, for 2 h. Slices were washed three times for 5, 10 and 60 min respectively in 0.05 M TBST (TBS containing 0.1% Tween 20). Then they were incubated at 4°C overnight with Alexa Fluor® 488-conjugated streptavidin (Invitrogen; dilution 1:1000 with TBST) and washed with TBST. Images were obtained with a confocal laser scanning microscope (SP2 1P-FCS, Leica). Usually the cells were located 50-100 μm from the slice surface. The somatic area and the number of the primary/secondary dendrites were counted by a rater blind to the animal history. Cells with less than three primary dendrites or less than one pair of secondary dendrites were excluded. The somatic area was calculated using ImageJ (NIH), by manually outlining the soma.

**Data Acquisition and Analysis**

Data were collected either 2 days after CIW/CIE (**Fig. S1**) or ~3 weeks (22+/-1 days, **Figs. 1-5**) after 4 cycles of CIW/CIE. All results are shown as mean ± SEM. Each experiment was replicated in at least 3 rats (usually 2-4 cells were recorded from 4 rats per group) for electrophysiological analysis and 8 rats for behavioral tests. Sample size in *in vivo* experiments (**Figs. 1B-E, 5 and S1B-D**) and Western blot (**Fig. 2K-P**) is presented as animal number (i.e., “n”); sample size in electrophysiology experiments (**Figs. 1F-T, 2C-J, 3, 4 and S1E, F**) and imaging data (**Fig. 2Q-S**) is presented as m/n, where "m" refers to the number of cells examined and "n" refers to the number of rats. Statistical significance was assessed using two-way ANOVA (summarized results in **Figs. 1B-E, 2, and 5**) or two-way ANOVA with repeated measures (summarized results in **Figs. 1F-T, 3, 4** and **S1F**), followed by Bonferroni post-hoc tests, or Student’s t test (**Fig. S1 B-D, G**).

**FIGURE LEGENDS**

**Figure S1. Neither anxiety-like behavior nor MSN excitability in the NAcS was affected 2 days after adolescent CIE, compared to the data 2 days after adolescent CIW.**

**A**, Experimental timeline for **Fig. S1**.

**B-D**, Summarized results showing no difference of crossover latency (**D**, t_14_=0.3, p=0.78), average speed (**E**, t_14_=0.7, p=0.47), or total distance travel (**F**, t_14_=0.3, p=0.77) between rats 2 days after the last intubation water *vs*. ethanol. The cell number / animal number (i.e., m/n) (**C**) or animal number (i.e., n) (**D-F**) is shown in parentheses for each group.

**E**, Example traces showing action potentials elicited by 200 and 400 pA current injections in NAcS MSNs from rats 2 days after CIW (left in the panel) and CIE (right in the panel) during the adolescent stage. **F**, Summarized data showing no difference in excitability of NAcS MSNs from rats acutely withdrawn from adolescent CIW *vs*. adolescent CIE (CIW/CIE × I_inj_ interaction F_7,133_=0.7, p=0.67, cell based; F_7,42_=0.9, p=0.52, animal based).

Data were analyzed by Student’s t test (**B-D**) or two-way ANOVA with repeated measures, followed by Bonferroni post-tests (**F**).

**Figure S2**. Summarized data showing **no effects of** **SK channel agonist (1-EBIO) or antagonist (Apamin) microinjected into the DLS on adolescent CIE induced anxiety-like behavior (**Ado::CIW/Ado::CIE X ACSF/Apamin/1-EBIO interaction F_2,30_=0.1, p=0.94; Ado::CIW/Ado::CIE F_1,30_=25.3, p<0.01; ACSF/Apamin/1-EBIO F_2,30_=0.3, p=0.81**).**  The animal number is shown in parentheses for each group. Data were analyzed by two-way ANOVA.

**REFERENCES**

1 Saalfield J, Spear L. Consequences of repeated ethanol exposure during early or late adolescence on conditioned taste aversions in rats. Developmental cognitive neuroscience. 2015;16:174-82.

2 Willey AR, Anderson RI, Morales M, Ramirez RL, Spear LP. Effects of ethanol administration on corticosterone levels in adolescent and adult rats. Alcohol. 2012;46(1):29-36.

3 Seminowicz DA, Laferriere AL, Millecamps M, Yu JS, Coderre TJ, Bushnell MC. MRI structural brain changes associated with sensory and emotional function in a rat model of long-term neuropathic pain. NeuroImage. 2009;47(3):1007-14.

4 Padula AE, Griffin WC, 3rd, Lopez MF, Nimitvilai S, Cannady R, McGuier NS, et al. KCNN Genes that Encode Small-Conductance Ca2+-Activated K+ Channels Influence Alcohol and Drug Addiction. Neuropsychopharmacology : official publication of the American College of Neuropsychopharmacology. 2015;40(8):1928-39.

5 Walter JT, Alvina K, Womack MD, Chevez C, Khodakhah K. Decreases in the precision of Purkinje cell pacemaking cause cerebellar dysfunction and ataxia. Nature neuroscience. 2006;9(3):389-97.

6 Ma YY, Lee BR, Wang X, Guo C, Liu L, Cui R, et al. Bidirectional modulation of incubation of cocaine craving by silent synapse-based remodeling of prefrontal cortex to accumbens projections. Neuron. 2014;83(6):1453-67.

7 Ma YY, Wang X, Huang Y, Marie H, Nestler EJ, Schluter OM, et al. Re-silencing of silent synapses unmasks anti-relapse effects of environmental enrichment. Proceedings of the National Academy of Sciences of the United States of America. 2016;113(18):5089-94.

8 Ma YY, Cepeda C, Chatta P, Franklin L, Evans CJ, Levine MS. Regional and cell-type-specific effects of DAMGO on striatal D1 and D2 dopamine receptor-expressing medium-sized spiny neurons. ASN neuro. 2012;4(2).

9 Ma YY, Chu NN, Guo CY, Han JS, Cui CL. NR2B-containing NMDA receptor is required for morphine-but not stress-induced reinstatement. Experimental neurology. 2007;203(2):309-19.

10 Ma YY, Guo CY, Yu P, Lee DY, Han JS, Cui CL. The role of NR2B containing NMDA receptor in place preference conditioned with morphine and natural reinforcers in rats. Experimental neurology. 2006;200(2):343-55.
